# Baseline Extracellular HMGB-1 Levels Associated with Induction Chemotherapy Response in Adult Acute Myeloid Leukemia

**DOI:** 10.12688/f1000research.183970.1

**Published:** 2026-07-11

**Authors:** Resti Mulya Sari, Lyana Setiawan, Damai Santosa, Syarifah Dewi, Melva Louisa, Ikhwan Rinaldi, Noorwati Sutandyo

**Affiliations:** 1 Division of Hematology and Medical Oncology, Dharmais Cancer Hospital, Jakarta, Indonesia; 2 Department of Hematology and Cellular Therapy, Dharmais Cancer Hospital, Jakarta, Indonesia; 3 Division of Hematology and Medical Oncology, Department of Internal Medicine, Faculty of Medicine, Diponegoro University, Semarang, Indonesia; 4 Department of Pharmacology and Therapeutics, Faculty of Medicine, Indonesia University, Jakarta, Indonesia; 5 Department of Biochemistry and Molecular Biology, Faculty of Medicine, Indonesia University, Jakarta, Indonesia; 6 Division of Hematology and Medical Oncology, Cipto Mangunkusumo National General Hospital, Faculty of Medicine, Indonesia University, Jakarta, Indonesia

**Keywords:** Acute myeloid leukemia; HMGB-1; induction chemotherapy; complete remission; immunogenic cell death; biomarker; DAMP; treatment response.

## Abstract

**Background:**

Treatment response in acute myeloid leukemia (AML) is influenced by multiple biological and molecular mechanisms. High Mobility Group Box-1 (HMGB-1) is a multifunctional protein involved in inflammation, autophagy, apoptosis, and immunogenic cell death (ICD). Extracellular HMGB-1 released following chemotherapy-induced leukemia cell death may enhance anti-tumor immune responses. This study aimed to evaluate the association between baseline extracellular HMGB-1 protein levels and achievement of complete remission following induction chemotherapy in adult AML patients.

**Methods:**

A prospective cohort study was conducted at Dharmais National Cancer Center, Jakarta, Indonesia. Adult patients with newly diagnosed AML who received standard D3A7 induction chemotherapy were enrolled consecutively. Baseline extracellular HMGB-1 protein levels were measured using enzyme-linked immunosorbent assay (ELISA) before chemotherapy initiation. Complete remission was assessed by bone marrow examination after induction chemotherapy. Receiver operating characteristic (ROC) curve analysis was used to determine the optimal HMGB-1 cut-off value. Associations between HMGB-1 levels and complete remission were evaluated using relative risks (RRs) with 95% confidence intervals (CIs).

**Results:**

Seventy-one AML patients were included in the final analysis. The median age at diagnosis was 39 years, and 52.1% were female. Complete remission was achieved in 40 patients (56.3%). Patients with low baseline extracellular HMGB-1 protein levels had a significantly lower probability of achieving complete remission than those with high HMGB-1 levels (RR 0.620; 95% CI 0.394–0.975; p = 0.038), corresponding to an approximately 38% reduction in remission likelihood. Among the evaluated biomolecular markers, extracellular HMGB-1 was the only biomarker significantly associated with complete remission.

**Conclusion:**

Low baseline extracellular HMGB-1 protein levels are associated with a lower probability of achieving complete remission following induction chemotherapy in adult AML patients. Extracellular HMGB-1 may serve as a potential biomolecular marker of treatment response in AML.

## Introduction

Acute Myeloblastic Leukemia (AML) is the most common form of acute leukemia found in the adult population. Based on data from Dharmais Cancer Hospital, Indonesia (2003–2007), AML accounting for 63.74% of acute leukemia cases with the highest proportion of patients was at 35 years of age (17.78%) which is considered as a productive age group. Treatment response in AML is influenced by multiple biological and molecular factors, including mechanisms related to DNA damage response, apoptosis, autophagy, and immunogenic cell death.

A combination of nucleoside analog and anthracyclines (D3A7 protocol) remains the standard induction chemotherapy for AML. ^1^ Cytarabine and daunorubicin induce DNA damage and apoptosis in leukemia cells through disruption of DNA replication and induction of double-strand DNA breaks. In addition to direct cytotoxicity, chemotherapy-induced leukemia cell death may activate immune-related signaling pathways through the release of intracellular molecules into the extracellular environment. ^2^ ^–^ ^4^

Several biomolecular factors involved in the DNA damage response (DDR) pathway have been associated with chemotherapy response in AML, including microRNA-181 family expression and activation of ATM and ATR proteins. ^2^ ^–^ ^7^ However, increasing evidence suggests that extracellular damage-associated molecular pattern (DAMP) molecules may also play an important role in determining treatment response.

High Mobility Group Box-1 (HMGB1) is a non-histone nuclear protein that functions as a multifunctional regulator involved in inflammation, autophagy, apoptosis, and tumor progression. Extracellular HMGB-1 released following cytotoxic chemotherapy acts as a damage-associated molecular pattern (DAMP) molecule capable of activating immunogenic cell death (ICD) pathways through Toll-like receptor (TLR) and receptor for advanced glycation end products (RAGE) signaling. ^2^ ^–^ ^4^ Activation of these pathways may enhance anti-tumor immune responses and contribute to chemotherapy sensitivity in AML.

This study aimed to evaluate the association between baseline extracellular HMGB-1 protein levels and achievement of complete remission following induction chemotherapy in adult acute myeloid leukemia patients.

## Methods

### Study Design and Patient Population

This study was conducted using a prospective cohort design at Dharmais National Cancer Center, Jakarta, Indonesia. Patients were recruited consecutively between March–December 2025. Eligible patients were aged >18 years and had newly diagnosed acute myeloid leukemia (AML) confirmed according to WHO diagnostic criteria 2022. Patients with acute promyelocytic leukemia (M3 subtype) and those with a history of myelodysplastic syndrome were excluded.

**Figure 1.**
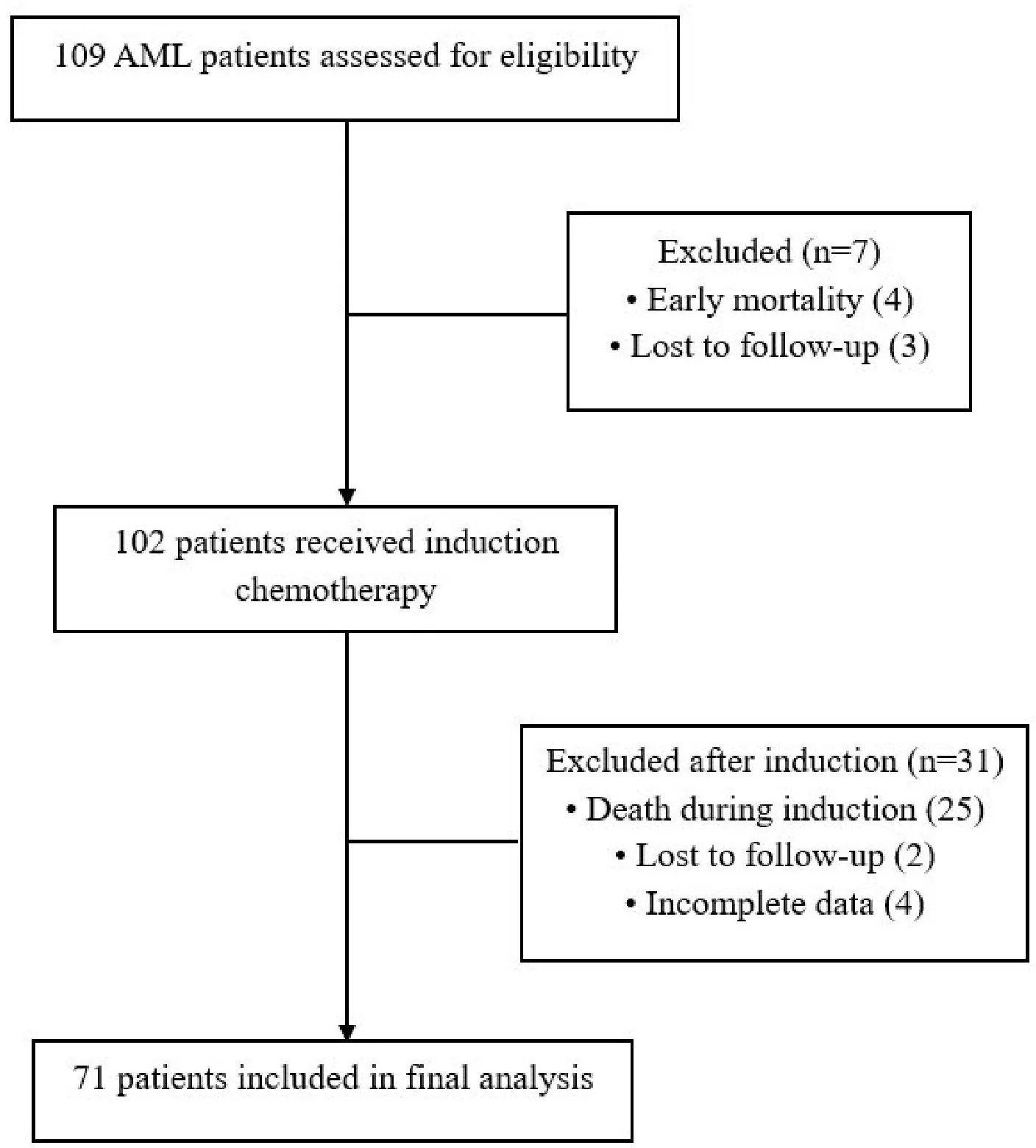
Flow diagram of patient recruitment and study population selection. A total of 109 adult patients with newly diagnosed acute myeloid leukemia (AML) were initially assessed for eligibility. Seven patients were excluded prior to induction chemotherapy due to early mortality (n = 4) and loss to follow-up (n = 3). Among the 102 patients who received induction chemotherapy, 31 patients were excluded from the final analysis due to death during induction chemotherapy (n = 25), loss to follow-up (n = 2), and incomplete clinical data (n = 4). Finally, 71 patients were included in the final analysis evaluating the association between extracellular HMGB-1 levels and induction chemotherapy response.

Initially, 109 adult patients with newly diagnosed AML were assessed for eligibility. Seven patients were excluded prior to induction chemotherapy due to early mortality (n = 4) and loss to follow-up (n = 3). Among the 102 patients who received induction chemotherapy, 31 patients were excluded from the final analysis due to death during induction chemotherapy (n = 25), loss to follow-up (n = 2), and incomplete clinical data (n = 4). Finally, 71 patients were included in the final analysis ( Figure 1).

### Sample Collection and Biomarker Measurement

Peripheral blood samples were collected at baseline prior to initiation of induction chemotherapy. Plasma was separated by centrifugation and stored at −80 °C until analysis.

Total RNA, including microRNAs, was extracted from peripheral blood samples using the miRNeasy Mini Kit (Qiagen, Hilden, Germany; Cat. No. 217004) according to the manufacturer’s instructions. Reverse transcription was performed using the miRCURY LNA RT Kit (Qiagen, Hilden, Germany; Cat. No. 339340). Expression levels of miR-181a-3p and miR-181b-5p were quantified using the miRCURY LNA miRNA PCR Assay (Qiagen, Hilden, Germany; Cat. No. 339306) in combination with the QIAcuity EG PCR Kit (Qiagen, Hilden, Germany; Cat. No. 250111). Absolute quantification was performed using the QIAcuity Digital PCR System (Qiagen, Hilden, Germany) according to the manufacturer’s protocol.

Serum ATM protein levels were measured using a Human ATM ELISA Kit (Colorimetric) (Novus Biologicals, Centennial, CO, USA; Cat. No. NBP2–69891). Serum ATR protein levels were measured using a Human Serine/Threonine-Protein Kinase ATR ELISA Kit (Abbexa Ltd., Cambridge, United Kingdom; Cat. No. abx555473). Serum HMGB-1 protein levels were measured using a Human HMGB1 ELISA Kit (Colorimetric) (Novus Biologicals, Centennial, CO, USA; Cat. No. NBP2–62766).

All ELISA measurements were performed according to the manufacturers’ instructions. Optical density was measured at 450 nm using a microplate reader, and protein concentrations were calculated from standard calibration curves generated using the supplied standards. One kit was used for ATM and HMGB-1 measurements, while two ATR ELISA kits were required to accommodate all study samples.

### Clinical and Morphologic Assessment

Patients who met the inclusion criteria received induction chemotherapy using the D3A7 regimen, daunorubicin 60–90 mg/m ^2^ for 3 days and cytarabine 100–200 mg/m ^2^ for seven days. Bone marrow aspiration evaluation was done at a minimum of day 21 after the initiation of induction chemotherapy.

### Statistical Analysis

All statistical analyses were performed using IBM SPSS Statistics for Windows, Version 26.0. Continuous variables were expressed as mean ± standard deviation (SD) or median with interquartile range (IQR), depending on data distribution. Categorical variables were presented as frequencies and percentages.

For categorical analyses, biomarker levels were categorized into high and low groups based on optimal cut-off values determined using receiver operating characteristic (ROC) curve analysis. ROC analysis was performed to identify cut-off values that best discriminated between patients who achieved and did not achieve complete remission following induction chemotherapy, based on the Youden index. The area under the curve (AUC), sensitivity, and specificity were calculated to evaluate the discriminatory performance of each biomarker.

Associations between clinical variables, biomarker levels, and complete remission status were evaluated using Pearson’s chi-square test or Fisher’s exact test, as appropriate. Relative risks (RRs) with 95% confidence intervals (CIs) were calculated to estimate the strength of associations. A two-sided p-value <0.05 was considered statistically significant.

## Results

### Patients’ Characteristics

Based on the study results, the median age at diagnosis of the patients was 39 years (range: 27–46), with the majority being female (52.11%) with the majority were classified as M2 subtype (47.89%). Forty patients (56.3%) were assessed as having achieved complete remission after induction chemotherapy ( Table 1).

**Table 1.** Patients’ Characteristics.

Patients’ Characteristics	N
Age at diagnosis (year, N = 71), median (IQ)	39 (27–46)
Sex, (N = 71, %)	
a. Male	34 (47.89)
b. Female	37 (52.11)
FAB subtypes, (N = 71, %)	
a. M0	1 (1.41)
b. M1	11 (15.49)
c. M2	34 (47.89)
d. M4	12 (16.90)
e. M5	12 (16.90)
f. M6	1 (1.41)
Complete remission after induction chemotherapy, (N = 71, %)	
a. Yes	40 (56.34)
b. No	31 (43.66)

IQ: Interquartile Range (Q1-Q3).

### Patients’ Characteristics Based on Treatment Results

Among the patients who achieved complete remission after induction chemotherapy, the majority were male (52.5%), with the most common FAB subtype is AML M2 (47.5%). The success of induction chemotherapy is influenced by several factors, both from the patient’s perspective and the disease characteristics, as shown in Table 2. One of the factors influencing the success of induction chemotherapy is organ function, including renal function. Statistical analysis showed that the median creatinine value was significantly associated with complete remission status (p = 0.023). These findings indicate that renal function plays a role in the success of induction chemotherapy ( Table 2).

**Table 2.** Clinical and Laboratory Characteristics According to Complete Remission Status.

Variable	Complete remission after induction chemotherapy		p value
	Yes (N = 40)	No (N = 31)	
Age at diagnosis (years), mean (SD)	36.43 (12.20)	37.23 (11.53)	0.780
Sex (%)			
a. Male	21 (52.5)	13 (41.9)	0.519
b. Female	19 (47.5)	18 (58.1)	
FAB Subtypes (%)			
a. M0	0 (0.0)	1 (3.2)	
b. M1	7 (17.5)	4 (12.9)	
c. M2	19 (47.5)	15 (48.4)	
d. M4	6 (15.0)	6 (19.4)	
e. M5	7 (17.5)	5 (16.1)	
f. M6	1 (2.5)	0 (0)	
Comorbidity			
a. Yes (≥ 1)	30 (75.0)	25 (80.6)	0.781
b. No	10 (25.0)	6 (19.4)	
Performance Status			
a. ECOG 0–1	34 (85.0)	26 (83.9)	1.000
b. ECOG >1	6 (15.0)	5 (16.1)	
Febrile Neutropenia			
a. Yes	36 (90.0)	26 (83.9)	0.490
b. No	4 (10.0)	5 (16.1)	
Initial percentage of blast cells in the bone marrow, mean (SD)	52.63 (17.97)	55.34 (22.72)	0.576
Duration of Febrile Neutropenia (days), mean (SD)	10.54 (6.65)	9.83 (6.64)	0.664
Haemoglobin (g/dL), mean (SD)	8.69 (1.89)	8.43 (2.93)	0.647
Leucocyte (u/L), median (IQ)	16950 (6400–48950)	15170 (6540–59820)	0.976
Platelet (x10 ^9^/L), median (IQ)	39 (23–132)	22 (15–60)	0.099
Creatinine (mg/dL), median (IQ)	0.8 (0.7–1.0)	0.7 (0.5–0.8)	0.023

SD: Standard deviation, IQ: Interquartile range (Q1-Q3).

### Association Between Baseline Biomolecular Markers and Complete Remission Status

Among the evaluated biomolecular markers, baseline extracellular HMGB-1 protein levels showed significant association with achievement of complete remission following induction chemotherapy. Patients with low baseline extracellular HMGB-1 levels had lower probability of achieving complete remission compared to patients with high HMGB-1 levels (RR 0.620, 95% CI: 0.394–0.975, p = 0.038). Other biomarkers including miRNA-181a-3p, miRNA-181b-5p, ATM, and ATR protein levels were not significantly associated with complete remission status ( Table 3).

**Table 3.** Association Between Baseline Biomolecular Markers and Complete Remission Following Induction Chemotherapy.

No	Variable	Complete remission after induction chemotherapy		RR (95% CI)	p
		Yes (N = 40)	No (N = 31)		
	**Biomolecular laboratory results, baseline**				
1	miRNA 181a-3p level, low (≤ 2.34 copies/uL)				
	a. Yes	18 (69.2)	8 (30.8)	1.416	
	b. No	22(48.9)	23 (51.1)	(0.955–2.099)	0.083
2	miRNA 181b-5p level, high (>197.5 copies/uL)				
	a. Yes	6 (40.0)	9 (60.0)	0.659	0.211
	b. No	34 (60.7)	22 (39.3)	(0.342–1.267)	
3	ATM protein level, high (>2.36 ng/ml)				
	a. Yes	3 (33.3)	6 (66.7)	0.931	0.731
	b. No	37 (59.7)	25 (40.3)	(0.617–1.403)	
4	ATR protein level, high (> 29.16 pg/ml)				
	a. Yes	25 (67.6)	12 (32.4)	1.532	0.057
	b. No	15 (44.1)	19 (55.9)	(0.987–2.376)	
5	HMGB-1 protein level, low (< 28.59 pg/ml)				
	a. Yes	14 (42.4)	19 (57.6)	0.6200	0.038
	b. No	26 (68.4)	12 (31.6)	(0.394–0.975)	

## Discussion

### Patients’ Characteristics

The median age at diagnosis of the study patients was 39 years (range: 27–46), with the majority being female (52.11%). Forty patients (56.3%) were assessed as having achieved complete remission after induction chemotherapy with the majority were male with ECOG performance status 0–1 and had comorbid diseases.

The European Leukemia Net (ELN) reported that 60–80% of AML patients undergoing induction chemotherapy achieved complete remission. ^8^ Lee (1996) ^9^ in South Korea reported that 61% of 132 patients receiving induction chemotherapy reached complete remission. Faleh (2015) ^10^ in Saudi Arabia treated 74 patients with induction chemotherapy, achieving remission in 84% of patients although 41% of them experienced relapse. ^10^ Other studies reporting treatment response in AML patients include Hadisantoso (2022) ^11^ who reported that 55.4% of patients achieved complete remission. Shireen (2022) ^12^ reported that 54% of patients reached complete remission following D3A7 induction chemotherapy. Meanwhile Zaidi (2022) ^13^ reported that 65.5% of 58 de novo AML patients achieved treatment response after induction chemotherapy.

### The role of HMGB-1 Protein Expression in Achieving Complete Remission

HMGB-1 protein is a non-histone nuclear protein included in the High Mobility Group Box subgroup, an important protein that has antiapoptotic and pro-autophagic properties. ^14^ HMGB-1 protein is a multifunctional molecule that is involved especially in inflammatory disorders and malignancies. ^15^ ^,^ ^16^ HMGB-1 contributes to leukemia progression through regulation of proliferation, survival signaling, inflammation, and resistance to cell death. ^16^ HMGB-1 protein has a higher expression level in bone marrow mononuclear cells of AML patients and contributes to the pathogenesis and progression of AML by inhibiting apoptosis, facilitating proliferation, and inducing inhibition of myeloid differentiation in AML cells. ^17^ ^–^ ^21^

Interestingly, among all evaluated biomolecular markers, only extracellular HMGB-1 demonstrated a significant association with complete remission following induction chemotherapy. This finding may suggest that biomarkers reflecting downstream immunogenic consequences of chemotherapy-induced leukemia cell death provide complementary information beyond conventional DNA damage response pathways. While ATM, ATR, and miR-181 are primarily involved in intracellular DNA damage signaling and leukemic cell biology, extracellular HMGB-1 may additionally reflect activation of host anti-leukemia immune responses through immunogenic cell death mechanisms, potentially explaining its stronger association with treatment outcome in the present study.

HMGB-1 functions as a “double-edged sword” that plays a role in both tumor promotion and inhibition during the development and progression of various cancers. ^22^ HMGB-1 protein as a damage-associated molecular pattern (DAMP) molecule can trigger immune and inflammatory responses through interactions with the Receptor for Advanced Glycation End Products (RAGE) and several members of Toll-like Receptors (TLR2 and TLR4). ^15^ ^,^ ^21^ HMGB-1 has also been found to be a key regulator of various cell death and signaling pathways and is involved in drug resistance by mediating cell autophagy and apoptosis, ferroptosis, pyroptosis, and various other signaling pathways. In addition, HMGB-1 is regulated by various non-coding RNAs (ncRNAs), such as microRNAs, long non-coding RNAs, and circular RNAs, which also play a role in drug resistance. ^22^ ^–^ ^24^

The dual biological role of HMGB-1 may explain the conflicting findings reported across malignancies. Intracellular HMGB-1 has been associated with autophagy-mediated chemoresistance, tumor survival, and inhibition of apoptosis. ^19^ ^,^ ^20^ ^,^ ^22^ ^,^ ^25^ In contrast, extracellular HMGB-1 released following cytotoxic chemotherapy may function as a damage-associated molecular pattern (DAMP) molecule that promotes immunogenic cell death and anti-tumor immune activation. ^2^ ^–^ ^4^ Therefore, extracellular HMGB-1 measured in this study may reflect effective chemotherapy-induced leukemia cell damage rather than intrinsic tumor aggressiveness.

In this study, low baseline extracellular HMGB-1 protein levels were significantly associated with a lower probability of achieving complete remission following induction chemotherapy (RR 0.620, 95% CI: 0.394–0.975, p = 0.038). Patients who achieved complete remission were more likely to have high baseline extracellular HMGB-1 protein levels than those who did not achieve remission. These findings suggest that low extracellular HMGB-1 levels may be associated with lower chemotherapy sensitivity and reduced induction treatment response in AML patients.

**Figure 2.**
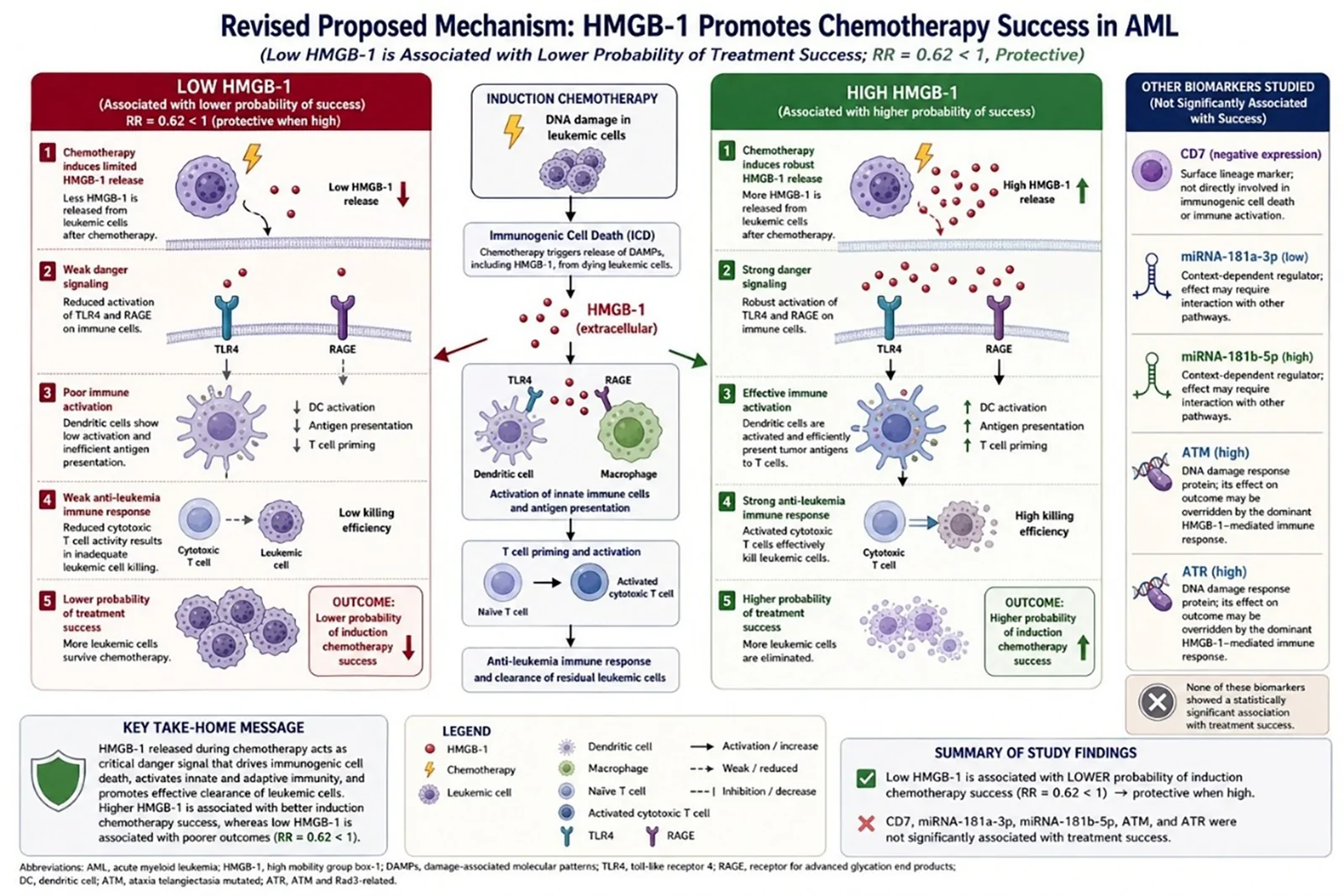
Proposed mechanistic model of extracellular HMGB-1–mediated immunogenic cell death (ICD) following induction chemotherapy in acute myeloid leukemia (AML). Induction chemotherapy using daunorubicin and cytarabine induces leukemia cell death and promotes extracellular release of HMGB-1 as a damage-associated molecular pattern (DAMP) molecule. Higher extracellular HMGB-1 levels are associated with stronger activation of Toll-like receptor 4 (TLR4) and receptor for advanced glycation end products (RAGE) signaling pathways on immune cells, leading to dendritic cell activation, enhanced antigen presentation, T-cell priming, and increased anti-leukemia immune response. These mechanisms may contribute to higher probability of achieving complete remission following induction chemotherapy. In contrast, lower extracellular HMGB-1 levels are associated with weaker immune activation and lower probability of treatment success. Other evaluated biomarkers including CD7, miRNA-181a-3p, miRNA-181b-5p, ATM, and ATR were not significantly associated with induction chemotherapy response in this study.

Chemotherapy-induced leukemia cell death may generate immunogenic signals that contribute not only to direct cytotoxicity but also to activation of anti-leukemia immune responses. During immunogenic cell death (ICD), dying leukemia cells release danger-associated molecular pattern (DAMP) molecules, including extracellular HMGB-1, into the tumor microenvironment. ^26^ ^–^ ^28^ Extracellular HMGB-1 subsequently interacts with Toll-like receptor 4 (TLR4) and receptor for advanced glycation end products (RAGE) expressed on antigen-presenting cells, particularly dendritic cells, resulting in dendritic cell maturation, enhanced antigen presentation, and activation of cytotoxic T-cell responses against residual leukemia cells. ^15^ ^,^ ^23^ Experimental studies have demonstrated that RAGE signaling contributes to immune activation, inflammatory signaling, and autophagy-related pathways, suggesting that HMGB1–RAGE interactions may represent an important mechanism linking chemotherapy-induced leukemia cell death with subsequent anti-tumor immune responses. ^23^ Therefore, extracellular HMGB-1 may not only represent a passive biomarker of leukemia cell death, but may also actively participate in immunologic processes contributing to chemotherapy effectiveness in AML ( Figure 2).

The findings of this study support the potential role of extracellular HMGB-1 as a clinically accessible biomolecular marker associated with induction chemotherapy response in AML patients. Although the exact mechanistic relationship between HMGB-1 signaling and chemotherapy sensitivity requires further investigation, the present study provides preliminary translational evidence supporting the role of HMGB-1-mediated immunogenic cell death in AML treatment response.

This study has several limitations. First, it was conducted in a single-center setting with a relatively modest sample size, particularly within specific FAB subgroups, which may limit the generalizability of the findings. Second, biomolecular examinations were performed only once prior to induction chemotherapy, and serial HMGB-1 measurements were not available to evaluate dynamic changes during treatment. Third, cytogenetic and molecular risk stratification analyses were not performed due to technical limitations, and functional studies were not conducted to directly elucidate the mechanistic relationship between HMGB-1 signaling, immunogenic cell death, and treatment response. Fourth, this study focused on early treatment response as reflected by complete remission following induction chemotherapy, whereas longer-term clinical outcomes such as relapse-free survival and overall survival were not evaluated.

In addition, the discriminatory performance of individual biomarkers may be limited when used in isolation, suggesting that future studies should explore integrated biomarker models to improve predictive accuracy. Nevertheless, this study was designed to evaluate the potential role of extracellular HMGB-1 as an accessible biomolecular marker that may complement existing prognostic approaches, particularly in resource-limited settings. Larger multicenter prospective studies incorporating molecular profiling, longitudinal HMGB-1 assessment, survival outcomes, and mechanistic validation are needed to confirm these findings and further define their clinical applicability.

## Conclusion

Low baseline extracellular HMGB-1 protein levels are associated with lower probability of achieving complete remission following induction chemotherapy in adult AML patients. These findings support the potential role of extracellular HMGB-1 as a biomolecular marker associated with chemotherapy-induced immunogenic cell death and treatment response in AML.

## Ethical considerations

This study was conducted in accordance with the principles of the Declaration of Helsinki. The parent prospective study from which this manuscript was derived received ethical approval from the Medical Research Ethics Committee of the Faculty of Medicine, Universitas Indonesia – Dr. Cipto Mangunkusumo National General Hospital (Approval No. KET-104/UN2.F1/ETIK/PPM.00.02/2025; approved on 7 February 2025).

As the study was conducted collaboratively across multiple study sites, including Dharmais National Cancer Center Hospital and Dr. Cipto Mangunkusumo National General Hospital, the research was also registered and acknowledged by the Health Research Ethics Committee of Dharmais Cancer Hospital (Notification No. DP.04.03/11.7/076/2025; dated 4 March 2025). The present manuscript represents a secondary biomarker analysis derived from the approved parent study. Written informed consent was obtained from all participants prior to enrolment.

## Data Availability

The datasets generated and analysed during the current study contain individual-level clinical and laboratory information from patients with acute myeloid leukemia. Public deposition of these data is restricted due to ethical and privacy considerations and in accordance with the conditions approved by the Health Research Ethics Committee of Dr. Cipto Mangunkusumo National General Hospital, Faculty of Medicine Universitas Indonesia. The Ethics Committee approved the study on the condition that participant confidentiality and privacy are protected. Therefore, raw patient-level data cannot be made publicly available. Researchers who wish to access de-identified data for legitimate academic purposes may submit a written request to the corresponding author ( restimulyasari@gmail.com). Requests will be reviewed by the corresponding author and the relevant institutional authorities. Access may be granted following ethical review, execution of a data-sharing agreement, and confirmation that the proposed use is consistent with the original informed consent and applicable regulations.
