# Baseline Extracellular HMGB-1 Levels Associated with Induction Chemotherapy Response in Adult Acute Myeloid Leukemia

**DOI:** 10.12688/f1000research.183970.2

**Published:** 2026-08-18

**Authors:** Resti Mulya Sari, Lyana Setiawan, Damai Santosa, Syarifah Dewi, Melva Louisa, Ikhwan Rinaldi, Noorwati Sutandyo

**Affiliations:** 1 Division of Hematology and Medical Oncology, Dharmais Cancer Hospital, Jakarta, Indonesia; 2 Department of Hematology and Cellular Therapy, Dharmais Cancer Hospital, Jakarta, Indonesia; 3 Division of Hematology and Medical Oncology, Department of Internal Medicine, Faculty of Medicine, Diponegoro University, Semarang, Indonesia; 4 Department of Pharmacology and Therapeutics, Faculty of Medicine, Indonesia University, Jakarta, Indonesia; 5 Department of Biochemistry and Molecular Biology, Faculty of Medicine, Indonesia University, Jakarta, Indonesia; 6 Division of Hematology and Medical Oncology, Cipto Mangunkusumo National General Hospital, Faculty of Medicine, Indonesia University, Jakarta, Indonesia

**Keywords:** Acute myeloid leukemia; HMGB-1; induction chemotherapy; complete remission; immunogenic cell death; biomarker; DAMP; treatment response.

## Abstract

**Background:**

Treatment response in acute myeloid leukemia (AML) is influenced by multiple biological and molecular mechanisms. High-mobility group box 1 (HMGB-1) is involved in inflammation, autophagy, apoptosis, and immunogenic cell death. This study evaluated associations of baseline circulating miR-181a-3p, miR-181b-5p, ATM, ATR, and HMGB-1 with successful induction response in adults with newly diagnosed AML.

**Methods:**

This prospective cohort study was conducted at two referral hospitals in Jakarta, Indonesia. Adults with newly diagnosed AML receiving standard D3A7 induction chemotherapy were enrolled consecutively. Baseline miR-181a-3p and miR-181b-5p were quantified using digital polymerase chain reaction, whereas ATM, ATR, and HMGB-1 were measured using enzyme-linked immunosorbent assays before chemotherapy. Successful induction response was assessed by clinical and bone marrow evaluation after induction. Receiver operating characteristic analysis determined an exploratory HMGB-1 cut-off. Associations were evaluated using relative risks (RRs) with 95% confidence intervals (CIs).

**Results:**

Among 102 patients initiating induction chemotherapy, 25 died during induction or early post-induction care. The biomarker-evaluable cohort comprised 71 patients, of whom 40 (56.3%) achieved a successful response. Baseline miR-181a-3p, miR-181b-5p, ATM, and ATR were not significantly associated with response. HMGB-1 yielded an area under the curve of 0.615 (95% CI, 0.480–0.749). The exploratory cut-off was 28.59 pg/mL, with 65.0% sensitivity and 61.3% specificity. Low HMGB-1 was associated with a lower probability of successful response (RR, 0.620; 95% CI, 0.394–0.975; p = 0.038). In the final clinical-biomolecular model, high HMGB-1 remained associated with successful response after adjustment for platelet count and ATR (adjusted RR, 1.666; 95% CI, 1.092–2.541; p = 0.018).

**Conclusion:**

Low baseline circulating HMGB-1 was associated with a lower probability of successful induction response in the biomarker-evaluable adult AML cohort. Given its modest discriminatory performance and internally derived cut-off, HMGB-1 should be considered an exploratory complementary biomarker requiring external validation and serial evaluation.

## Introduction

Acute myeloid leukemia (AML) is the most common form of acute leukemia in the adult population and represents a biologically and clinically heterogeneous hematologic malignancy. The burden of leukemia in Indonesia remains substantial. According to the Global Cancer Observatory estimates for 2024, leukemia accounted for approximately 21,912 new cases, 14,643 deaths, and 61,806 five-year prevalent cases in Indonesia. ^1^ However, these estimates include all leukemia subtypes because contemporary nationwide AML-specific incidence and prevalence data are not currently available. Indonesian evidence is therefore predominantly derived from hospital-based cohorts. Hadisantoso et al. identified 125 adults with newly diagnosed non-acute promyelocytic AML who received standard 7 + 3 induction chemotherapy at two Indonesian national referral hospitals between 2015 and 2019. Of these patients, 24 (19.2%) died before treatment-response evaluation, while 55.4% of the 101 evaluable patients achieved complete remission. The median age was 39 years, and AML M2 was the most frequent FAB subtype. ^2^ These findings provide relevant contemporary clinical data regarding intensively treated Indonesian patients with AML, although they should not be interpreted as national prevalence estimates.

Despite advances in diagnosis and treatment, AML remains associated with considerable early mortality, induction failure, relapse, and poor long-term survival. The overall five-year relative survival remains approximately one-third and is substantially lower among older patients. ^2^ Overall survival (OS) is generally defined as the interval from diagnosis or treatment initiation to death from any cause, whereas event-free survival (EFS) incorporates events such as failure to achieve remission, relapse, or death. AML outcomes are influenced by age, performance status, comorbidities, disease ontogeny, leukocyte and blast counts, cytogenetic and molecular abnormalities, treatment intensity, induction response, and measurable residual disease status. ^2^ ^,^ ^3^ Factors associated with the development of AML include increasing age, antecedent myelodysplastic or myeloproliferative neoplasms, inherited cancer-predisposition syndromes, previous cytotoxic chemotherapy or radiation exposure, benzene exposure, and cigarette smoking. ^2^

The 2022 European LeukemiaNet (ELN) recommendations classify AML into favorable-, intermediate-, and adverse-risk groups according to genetic abnormalities at diagnosis. ^3^ Favorable-risk AML includes RUNX1::RUNX1T1, CBFB::MYH11, mutated NPM1 without FLT3-ITD, and in-frame mutations affecting the basic leucine zipper region of CEBPA. Intermediate-risk AML includes FLT3-ITD-positive disease, MLLT3::KMT2A, and abnormalities not classified as favorable or adverse. Adverse-risk AML includes complex or monosomal karyotypes, TP53 mutation, selected high-risk rearrangements, and mutations associated with myelodysplasia-related disease. ^3^ ELN risk classification is associated with remission, relapse, EFS, and OS and is therefore important for contemporary AML prognostication. ^4^ However, comprehensive cytogenetic and molecular profiling is not routinely available for all patients in resource-limited clinical settings.

A combination of nucleoside analog and anthracyclines (D3A7 protocol) remains the standard induction chemotherapy for AML. ^5^ Cytarabine and daunorubicin induce DNA damage and apoptosis in leukemia cells through disruption of DNA replication and induction of double-strand DNA breaks. In addition to direct cytotoxicity, chemotherapy-induced leukemia cell death may activate immune-related signaling pathways through the release of intracellular molecules into the extracellular environment. ^6^ ^,^ ^7^

Several biomolecular factors involved in the DNA damage response (DDR) pathway have been associated with chemotherapy response in AML, including microRNA-181 family expression and activation of ATM and ATR proteins. ^6^ ^–^ ^11^ However, increasing evidence suggests that extracellular damage-associated molecular pattern (DAMP) molecules may also play an important role in determining treatment response.

High Mobility Group Box-1 (HMGB1) is a non-histone nuclear protein that functions as a multifunctional regulator involved in inflammation, autophagy, apoptosis, and tumor progression. Extracellular HMGB-1 released following cytotoxic chemotherapy acts as a damage-associated molecular pattern (DAMP) molecule capable of activating immunogenic cell death (ICD) pathways through Toll-like receptor (TLR) and receptor for advanced glycation end products (RAGE) signaling. ^6^ ^–^ ^8^ Activation of these pathways may enhance anti-tumor immune responses and contribute to chemotherapy sensitivity in AML.

This study aimed to evaluate the associations of baseline circulating miR-181a-3p, miR-181b-5p, ATM, ATR, and HMGB-1 levels measured before induction chemotherapy with successful induction response in adults with newly diagnosed AML. Baseline HMGB-1 was interpreted as a circulating biomarker reflecting underlying leukemic and host inflammatory biology rather than as a direct measurement of HMGB-1 released in response to chemotherapy.

## Methods

### Study Design and Patient Population

This study was conducted using a prospective cohort design at Dharmais National Cancer Center, Jakarta, Indonesia. Patients were recruited consecutively between March–December 2025. Eligible patients were aged >18 years and had newly diagnosed acute myeloid leukemia (AML) confirmed according to WHO diagnostic criteria 2022. Patients with acute promyelocytic leukemia corresponding to the M3 FAB subtype, a previous diagnosis of myelodysplastic syndrome, severe clinical conditions that precluded intensive chemotherapy, death before completion of induction chemotherapy, unavailable post-induction bone marrow evaluation, or non-evaluable biomarker laboratory results were excluded from the biomarker-evaluable analysis. Pediatric patients were not included because the parent study was specifically designed for adults treated within adult hematology-oncology services. Pediatric AML differs from adult AML in its cytogenetic and molecular landscape, treatment protocols, chemotherapy dose intensity, supportive-care requirements, treatment-related toxicity, and long-term outcomes. Combining pediatric and adult patients could therefore introduce substantial biological and therapeutic heterogeneity.

### Patient Selection

**Figure 1.**
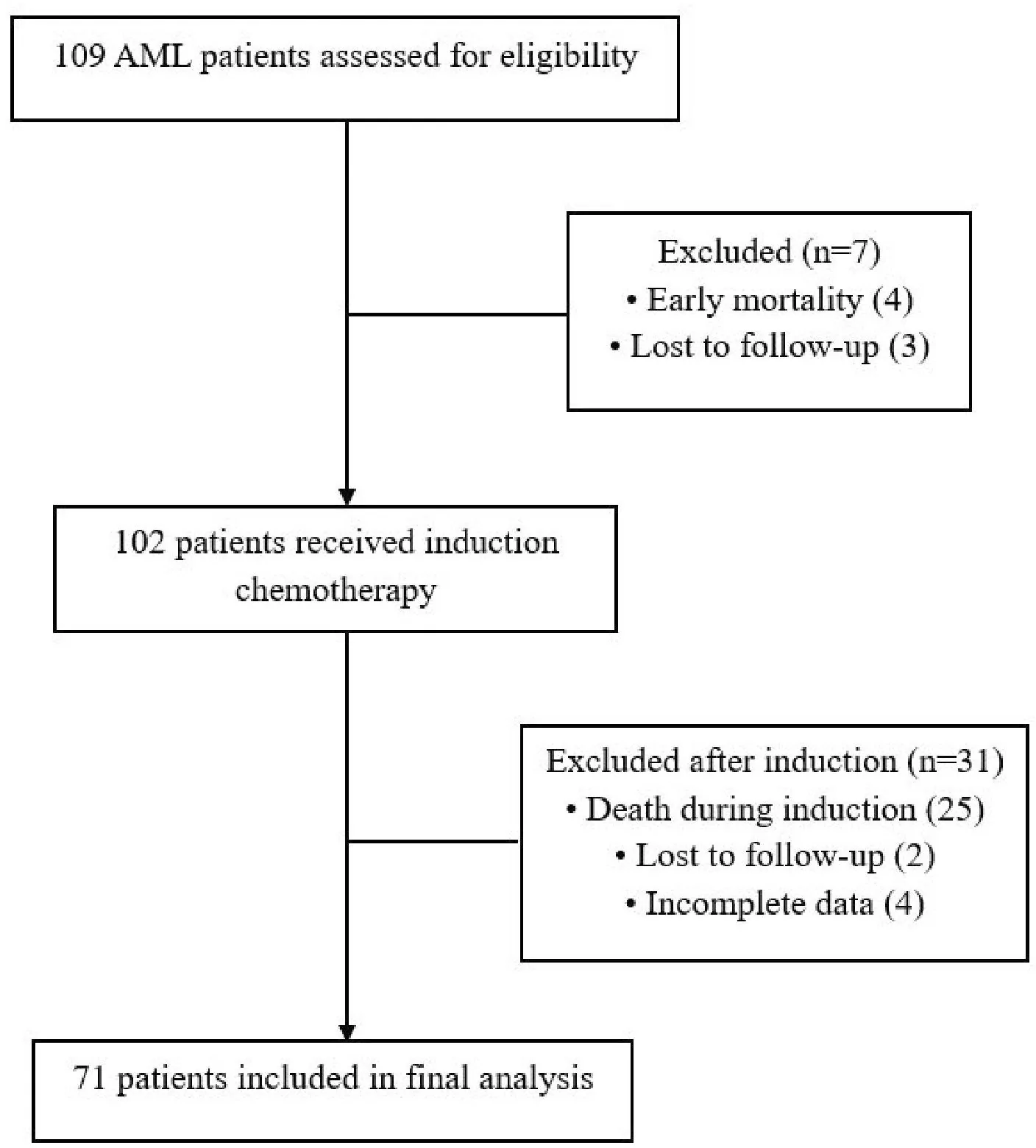
Flow diagram of patient recruitment and study population selection. A total of 109 adult patients with newly diagnosed acute myeloid leukemia (AML) were initially assessed for eligibility. Seven patients were excluded prior to induction chemotherapy due to early mortality (n = 4) and loss to follow-up (n = 3). Among the 102 patients who received induction chemotherapy, 31 patients were excluded from the final analysis due to death during induction chemotherapy (n = 25), loss to follow-up (n = 2), and incomplete clinical data (n = 4). Finally, 71 patients were included in the final analysis evaluating the association between extracellular HMGB-1 levels and induction chemotherapy response.

Initially, 109 adult patients with newly diagnosed AML were assessed for eligibility. Seven patients were excluded prior to induction chemotherapy due to early mortality (n = 4) and loss to follow-up (n = 3). Among the 102 patients who received induction chemotherapy, 31 patients were excluded from the final analysis due to death during induction chemotherapy (n = 25), loss to follow-up (n = 2), and incomplete clinical data (n = 4). Finally, 71 patients were included in the final analysis ( Figure 1).

The 71-patient cohort should be interpreted as an evaluable-patient biomarker cohort rather than the entire population initiating induction chemotherapy. Induction mortality among the 102 treated patients is reported separately to provide a transparent description of treatment attrition.

### Sample-size Calculation

The sample size of the parent prospective cohort was calculated using a single diagnostic-sensitivity formula:

$$\text{Nsen}={\mathrm{Z\alpha}}^{2}\times \mathrm{Sen}\left(1-\mathrm{Sen}\right)/\left(d^{2}\times P\right)$$

The calculation assumed an expected biomarker sensitivity of 70%, an anticipated prevalence of successful induction response of 55%, a precision of 15%, and a two-sided alpha level of 5%, corresponding to a Zα value of 1.96. The minimum calculated sample size was 66 participants. Ultimately, 71 patients had complete induction-response and biomarker data and were included in the final analysis, thereby exceeding the minimum required sample size.

### Sample Collection and Biomarker Measurement

Peripheral blood samples were collected at baseline before initiation of induction chemotherapy. Plasma or serum was separated according to the requirements of each biomarker assay and stored at −80°C until analysis.

Total RNA, including microRNAs, was extracted from peripheral blood samples using the miRNeasy Mini Kit (Qiagen, Hilden, Germany; Cat. No. 217004) according to the manufacturer’s instructions. Reverse transcription was performed using the miRCURY LNA RT Kit (Qiagen, Hilden, Germany; Cat. No. 339340). Expression levels of miR-181a-3p and miR-181b-5p were quantified using the miRCURY LNA miRNA PCR Assay (Qiagen, Hilden, Germany; Cat. No. 339306) in combination with the QIAcuity EG PCR Kit (Qiagen, Hilden, Germany; Cat. No. 250111). Absolute quantification was performed using the QIAcuity Digital PCR System (Qiagen, Hilden, Germany) according to the manufacturer’s protocol.

Serum ATM protein levels were measured using a Human ATM ELISA Kit (Colorimetric) (Novus Biologicals, Centennial, CO, USA; Cat. No. NBP2–69891). Serum ATR protein levels were measured using a Human Serine/Threonine-Protein Kinase ATR ELISA Kit (Abbexa Ltd., Cambridge, United Kingdom; Cat. No. abx555473). Serum HMGB-1 protein levels were measured using a Human HMGB1 ELISA Kit (Colorimetric) (Novus Biologicals, Centennial, CO, USA; Cat. No. NBP2–62766).

All ELISA measurements were performed according to the manufacturers’ instructions. Optical density was measured at 450 nm using a microplate reader, and protein concentrations were calculated from standard calibration curves generated using the supplied standards. One kit was used for ATM and HMGB-1 measurements, while two ATR ELISA kits were required to accommodate all study samples.

### Clinical and Morphologic Assessment

Patients who met the inclusion criteria received induction chemotherapy using the D3A7 regimen, daunorubicin 60–90 mg/m ^2^ for 3 days and cytarabine 100–200 mg/m ^2^ for seven days. Bone marrow aspiration evaluation was done at a minimum of day 21 after the initiation of induction chemotherapy.

### Statistical Analysis

Continuous variables were summarized as mean (standard deviation) or median (interquartile range), as appropriate, and categorical variables as frequencies and percentages. Exploratory biomarker cut-offs were determined using receiver operating characteristic curve analysis and the Youden index. HMGB-1 was categorized as low (≤28.59 pg/mL) or high (>28.59 pg/mL). Because the cut-off was derived and evaluated in the same cohort, the corresponding categorical analyses were considered exploratory.

Associations between categorical variables and induction response were evaluated using Pearson’s chi-square or Fisher’s exact test, as appropriate, and reported as relative risks with 95% confidence intervals. Continuous variables were compared using appropriate parametric or non-parametric tests. Spearman’s rank correlation was used to evaluate the relationships between baseline HMGB-1 and bone marrow and peripheral blood blast percentages, miR-181a-3p, miR-181b-5p, ATM, and ATR.

Variables with p < 0.25 in bivariable analysis were considered for multivariable analysis using backward elimination. The initial clinical-biomolecular model included platelet count >22,500/μL, serum creatinine >0.74 mg/dL, HMGB-1 > 28.59 pg/mL, and ATR >29.16 pg/mL. Serum creatinine was removed during backward elimination, leaving platelet count, HMGB-1, and ATR in the final model. Adjusted relative risks with 95% confidence intervals were reported, and multicollinearity was assessed using variance inflation factors. All tests were two-sided, with p < 0.05 considered statistically significant. Analyses were performed using IBM SPSS Statistics version 26.0.

## Results

### Patients’ Characteristics

The median age at diagnosis was 39 years (IQR, 27–46 years), and 37 patients (52.1%) were female. AML M2 was the most frequent FAB subtype, accounting for 34 patients (47.9%). Forty patients (56.3%) achieved a successful induction response, whereas 31 patients (43.7%) did not. The patients’ characteristics are presented in Table 1.

**Table 1.** Patients’ Characteristics.

Patients’ Characteristics	N
Age at diagnosis (year, N = 71), median (IQ)	39 (27–46)
Sex, (N = 71, %)	
a. Male	34 (47.89)
b. Female	37 (52.11)
FAB subtypes, (N = 71, %)	
a. M0	1 (1.41)
b. M1	11 (15.49)
c. M2	34 (47.89)
d. M4	12 (16.90)
e. M5	12 (16.90)
f. M6	1 (1.41)
Successful induction response after induction chemotherapy, (N = 71, %)	
a. Yes	40 (56.34)
b. No	31 (43.66)

IQ: Interquartile Range (Q1-Q3).

### Patients’ Characteristics Based on Treatment Results

Among the 40 patients who achieved a successful induction response, 21 (52.5%) were male, 34 (85.0%) had an ECOG performance status of 0–1, and 30 (75.0%) had at least one comorbidity. AML M2 was the most frequent FAB subtype in both the successful- and unsuccessful-response groups, accounting for 47.5% and 48.4% of patients, respectively.

There were no significant differences between the successful- and unsuccessful-response groups in age, sex, FAB subtype, comorbidity, ECOG performance status, febrile neutropenia, duration of febrile neutropenia, baseline bone marrow blast percentage, hemoglobin concentration, leukocyte count, or platelet count. Serum creatinine differed significantly between the two groups, with a median concentration of 0.80 mg/dL (IQR, 0.70–1.00) in the successful-response group and 0.70 mg/dL (IQR, 0.50–0.80) in the unsuccessful-response group (p = 0.023). Complete comparisons are presented in Table 2.

**Table 2.** Clinical and Laboratory Characteristics According to Successful Induction Response Status.

Variable	Successful induction response after induction chemotherapy		p value
	Yes (N = 40)	No (N = 31)	
Age at diagnosis (years), mean (SD)	36.43 (12.20)	37.23 (11.53)	0.780
Sex (%)			
a. Male	21 (52.5)	13 (41.9)	0.519
b. Female	19 (47.5)	18 (58.1)	
FAB Subtypes (%)			
a. M0	0 (0.0)	1 (3.2)	
b. M1	7 (17.5)	4 (12.9)	
c. M2	19 (47.5)	15 (48.4)	
d. M4	6 (15.0)	6 (19.4)	
e. M5	7 (17.5)	5 (16.1)	
f. M6	1 (2.5)	0 (0)	
Comorbidity			
a. Yes (≥ 1)	30 (75.0)	25 (80.6)	0.781
b. No	10 (25.0)	6 (19.4)	
Performance Status			
a. ECOG 0–1	34 (85.0)	26 (83.9)	1.000
b. ECOG >1	6 (15.0)	5 (16.1)	
Febrile Neutropenia			
a. Yes	36 (90.0)	26 (83.9)	0.490
b. No	4 (10.0)	5 (16.1)	
Initial percentage of blast cells in the bone marrow, mean (SD)	52.63 (17.97)	55.34 (22.72)	0.576
Duration of Febrile Neutropenia (days), mean (SD)	10.54 (6.65)	9.83 (6.64)	0.664
Haemoglobin (g/dL), mean (SD)	8.69 (1.89)	8.43 (2.93)	0.647
Leucocyte (u/L), median (IQ)	16950 (6400–48950)	15170 (6540–59820)	0.976
Platelet (x10 ^9^/L), median (IQ)	39 (23–132)	22 (15–60)	0.099
Creatinine (mg/dL), median (IQ)	0.8 (0.7–1.0)	0.7 (0.5–0.8)	0.023

SD: Standard deviation, IQ: Interquartile range (Q1-Q3).

### Association Between Baseline Biomolecular Markers and Induction Treatment Response

Among the evaluated baseline biomolecular markers, only circulating HMGB-1 was significantly associated with successful induction response. Low baseline HMGB-1 levels, defined as ≤28.59 pg/mL, were observed in 33 patients. Of these patients, 14 (42.4%) achieved a successful response and 19 (57.6%) did not. Among the 38 patients with high baseline HMGB-1 levels, 26 (68.4%) achieved a successful response and 12 (31.6%) did not.

**Figure 2.**
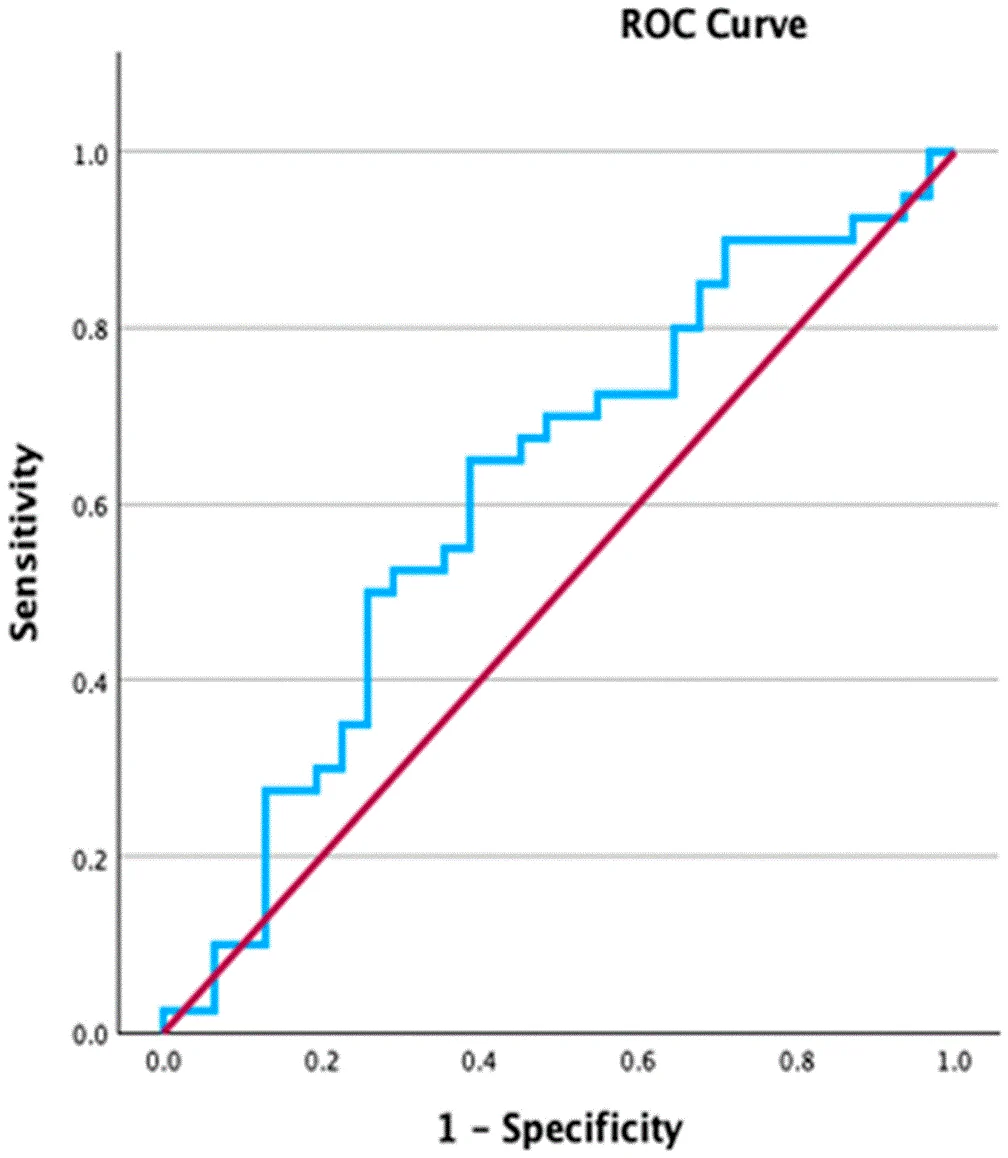
Receiver operating characteristic curve of baseline circulating HMGB-1 for predicting successful induction response. The AUC was 0.615 (95% CI, 0.480–0.749). The optimal cut-off value determined using the Youden index was 28.59 pg/mL, with a sensitivity of 65.0% and a specificity of 61.3%.

The ROC analysis of baseline circulating HMGB-1 for predicting successful induction response yielded an AUC of 0.615 (95% CI 0.480–0.749). The Youden index identified an optimal cut-off value of 28.59 pg/mL, with a sensitivity of 65.0% and a specificity of 61.3%. HMGB-1 concentrations ≤28.59 pg/mL were categorized as low, whereas concentrations >28.59 pg/mL were categorized as high ( Figure 2).

Patients with low baseline HMGB-1 had a lower probability of achieving a successful induction response than patients with high HMGB-1 levels (RR 0.620; 95% CI 0.394–0.975; p = 0.038), corresponding to an approximately 38% lower probability of successful response.

Baseline miR-181a-3p, miR-181b-5p, ATM, and ATR categories were not significantly associated with induction response. The association for high ATR approached but did not reach the predefined level of statistical significance (RR 1.532; 95% CI 0.987–2.376; p = 0.057). The results of the biomarker analyses are presented in Table 3.

**Table 3.** Association Between Baseline Biomolecular Markers and Successful Induction Response Following Induction Chemotherapy.

No	Variable	Successful induction response after induction chemotherapy		RR (95% CI)	p
		Yes (N = 40)	No (N = 31)		
	**Biomolecular laboratory results, baseline**				
1	miRNA 181a-3p level, low (≤2.34 copies/uL)				
	a. Yes	18 (69.2)	8 (30.8)	1.416	
	b. No	22(48.9)	23 (51.1)	(0.955–2.099)	0.083
2	miRNA 181b-5p level, high (>197.5 copies/uL)				
	a. Yes	6 (40.0)	9 (60.0)	0.659	0.211
	b. No	34 (60.7)	22 (39.3)	(0.342–1.267)	
3	ATM protein level, high (>2.36 ng/ml)				
	a. Yes	3 (33.3)	6 (66.7)	0.931	0.731
	b. No	37 (59.7)	25 (40.3)	(0.617–1.403)	
4	ATR protein level, high (>29.16 pg/ml)				
	a. Yes	25 (67.6)	12 (32.4)	1.532	0.057
	b. No	15 (44.1)	19 (55.9)	(0.987–2.376)	
5	HMGB-1 protein level, low (< 28.59 pg/ml)				
	a. Yes	14 (42.4)	19 (57.6)	0.6200	0.038
	b. No	26 (68.4)	12 (31.6)	(0.394–0.975)	

### Association between HMGB-1 and Clinicopathological Characteristics

The correlations between baseline circulating HMGB-1 and the evaluated morphologic and biomolecular characteristics are presented in Table 4. Baseline HMGB-1 concentration was not significantly correlated with bone marrow blast percentage at diagnosis (Spearman’s r = −0.090; p = 0.457) or peripheral blood blast percentage (r = 0.118; p = 0.331). These findings indicate that baseline circulating HMGB-1 was not detectably associated with morphologic blast burden in this cohort.

**Table 4.** Correlations Between Baseline HMGB-1 Concentration and Morphologic and Biomolecular Characteristics.

Variable correlated with baseline HMGB-1	n	Spearman’s r	p-value
**Bone marrow blast percentage at diagnosis**	71	−0.090	0.457
**Peripheral blood blast percentage at diagnosis**	71	0.118	0.331
**Circulating miR-181a-3p**	71	−0.031	0.797
**Circulating miR-181b-5p**	71	−0.068	0.576
**ATM concentration**	71	−0.034	0.781
**ATR concentration**	71	0.148	0.217

HMGB-1: high-mobility group box 1; ATM: ataxia-telangiectasia mutated; ATR: ataxia-telangiectasia and Rad3-related. Correlations were evaluated using Spearman’s rank correlation.

No statistically significant correlations were identified between baseline HMGB-1 and circulating miR-181a-3p (r = −0.031; p = 0.797), miR-181b-5p (r = −0.068; p = 0.576), or ATM concentration (r = −0.034; p = 0.781). Baseline HMGB-1 was also not significantly correlated with ATR concentration observed in the cohort and included in the analysis (r = 0.148; p = 0.217; n = 71). Overall, the correlations were weak, and none of the evaluated morphologic or biomolecular characteristics demonstrated a statistically significant correlation with baseline circulating HMGB-1.

### Multivariable Analysis

Variables meeting the predefined criterion of p < 0.25 in the bivariable analysis were considered for the multivariable clinical-biomolecular model. The initial model included platelet count >22,500/μL, serum creatinine >0.74 mg/dL, high baseline HMGB-1 > 28.59 pg/mL, and high baseline ATR >29.16 pg/mL. In the initial model, platelet count (RR 1.926; 95% CI 1.149–3.229; p = 0.013) and high ATR (RR 1.534; 95% CI 1.042–2.257; p = 0.030) were significantly associated with successful induction response. The association with high HMGB-1 approached statistical significance (RR 1.529; 95% CI 0.993–2.354; p = 0.054), whereas serum creatinine was not significantly associated with response (RR 0.697; 95% CI 0.454–1.072; p = 0.101).

After serum creatinine was removed through backward elimination, platelet count >22,500/μL, high baseline HMGB-1, and high baseline ATR were retained in the final model. High baseline HMGB-1 was associated with a higher probability of successful induction response after adjustment for platelet count and ATR (adjusted RR 1.666; 95% CI 1.092–2.541; p = 0.018). Platelet count >22,500/μL (adjusted RR 1.947; 95% CI 1.151–3.293; p = 0.013) and high baseline ATR (adjusted RR 1.514; 95% CI 1.026–2.263; p = 0.037) were also associated with successful response. No relevant multicollinearity was identified among the variables included in the model. The findings of the multivariable biomarker analysis are presented in Table 5.

**Table 5.** Multivariable Clinical-Biomolecular Analysis of Factors Associated with Successful Induction Response.

Model and variable	Adjusted RR (95% CI)	p-value
**Initial model**		
**Platelet count > 22,500/μL**	1.926 (1.149–3.229)	0.013
**Serum creatinine > 0.74 mg/dL**	0.697 (0.454–1.072)	0.101
**High HMGB-1 > 28.59 pg/mL**	1.529 (0.993–2.354)	0.054
**High ATR > 29.16 pg/mL**	1.534 (1.042–2.257)	0.030
**Final model**		
**Platelet count > 22,500/μL**	1.947 (1.151–3.293)	0.013
**High HMGB-1 > 28.59 pg/mL**	1.666 (1.092–2.541)	0.018
**High ATR > 29.16 pg/mL**	1.514 (1.026–2.263)	0.037

RR: relative risk; CI: confidence interval; HMGB-1: high-mobility group box 1; ATR: ataxia-telangiectasia and Rad3-related.

## Discussion

### Patients’ Characteristics

The median age at diagnosis was 39 years (IQR, 27–46 years), and 52.1% of the patients were female. Forty patients (56.3%) achieved a successful induction response. Among these patients, most had an ECOG performance status of 0–1 and at least one comorbidity.

Previous studies have reported varying response rates following AML induction chemotherapy. Lee et al. reported complete remission in 61% of 132 patients receiving induction chemotherapy in South Korea. ^16^ Zahrani et al. reported remission in 84% of patients treated in Saudi Arabia, although 41% subsequently experienced relapse. ^17^ In Indonesia, Hadisantoso et al. reported complete remission in 55.4% of evaluable patients treated with standard 7 + 3 induction chemotherapy. ^2^ Shireen et al. reported complete remission in 54% of patients receiving standard 3 + 7 induction therapy. ^18^ Whereas Zaidi et al. reported a post-induction response rate of approximately 65.5%. ^19^ Differences among studies may be related to patient age, performance status, AML subtype, molecular risk, treatment intensity, supportive care, and response definitions.

In the present study, 25 of 102 patients who initiated induction chemotherapy died during induction or early post-induction care. These patients could not undergo standardized post-induction bone marrow evaluation and were therefore excluded from the final biomarker analysis. Consequently, the response rate reported among the 71 evaluable patients should not be interpreted as the response rate among all patients who initiated induction chemotherapy.

### The role of HMGB-1 Protein Expression in Achieving Complete Remission

HMGB-1 protein is a non-histone nuclear protein included in the High Mobility Group Box subgroup, an important protein that has antiapoptotic and pro-autophagic properties. ^20^ HMGB-1 protein is a multifunctional molecule that is involved especially in inflammatory disorders and malignancies. ^21^ ^,^ ^22^ HMGB-1 contributes to leukemia progression through regulation of proliferation, survival signaling, inflammation, and resistance to cell death. ^22^ HMGB-1 protein has a higher expression level in bone marrow mononuclear cells of AML patients and contributes to the pathogenesis and progression of AML by inhibiting apoptosis, facilitating proliferation, and inducing inhibition of myeloid differentiation in AML cells. ^23^ ^–^ ^27^

Among the evaluated baseline biomolecular markers, circulating HMGB-1 was significantly associated with successful induction response. Patients with low baseline HMGB-1 had a lower probability of successful response than those with high levels. This direction was consistent with the final clinical-biomolecular model, in which high baseline HMGB-1 remained associated with successful induction response after adjustment for platelet count and ATR. These findings suggest that baseline circulating HMGB-1 may provide complementary information regarding early treatment response alongside the clinical and biomolecular variables included in the model.

Baseline HMGB-1 was not significantly correlated with bone marrow or peripheral blood blast percentages, miR-181a-3p, miR-181b-5p, ATM, or ATR. Thus, circulating HMGB-1 was not detectably associated with morphologic blast burden or the other evaluated baseline biomolecular markers in this cohort. Nevertheless, the absence of statistically significant correlations does not establish biological independence because circulating HMGB-1 may originate from multiple cellular and tissue sources.

The direction of the association observed in this study appears to differ from that reported in several previous AML studies, in which higher HMGB-1 expression has generally been associated with more aggressive disease biology and chemotherapy resistance. This apparent discrepancy may partly reflect differences in the biological compartment and timing of HMGB-1 measurement. Many previous studies evaluated intracellular HMGB-1 or its expression in bone marrow cells, where HMGB-1 is associated with leukemic-cell survival, autophagy activation, inhibition of apoptosis, and chemoresistance. ^25^ ^,^ ^26^ ^,^ ^28^ ^,^ ^30^ ^,^ ^31^ In contrast, the present study measured circulating HMGB-1 in peripheral blood before induction chemotherapy, which may represent a different aspect of leukemic and host biology.

This context-dependent activity is consistent with the “double-edged sword” nature of HMGB-1. ^22^ ^,^ ^28^ Intracellular HMGB-1 may promote leukemic-cell survival and treatment resistance through regulation of autophagy, apoptosis, ferroptosis, pyroptosis, and DNA-damage-related signaling. Its extracellular activity is also not uniform but depends on its molecular form and redox state. Fully reduced HMGB-1 primarily exhibits chemotactic activity, disulfide HMGB-1 can induce cytokine signaling through the TLR4/MD-2 complex, whereas terminal oxidation may abolish these extracellular activities. ^12^ Therefore, a higher total circulating HMGB-1 concentration cannot automatically be interpreted as evidence of an immune-stimulatory or antitumor effect. ^21^ ^,^ ^27^

**Figure 3.**
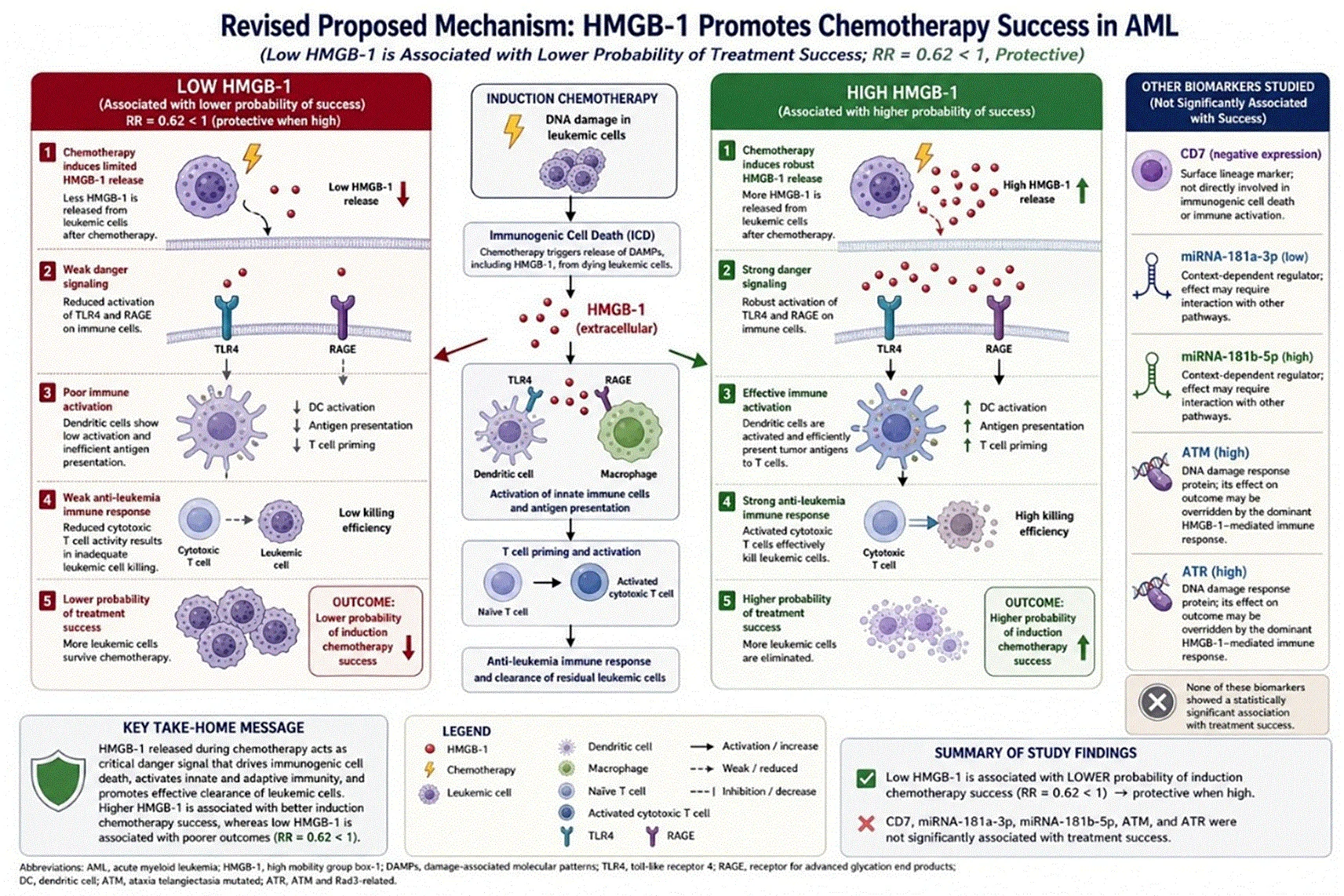
Conceptual Model Linking Baseline Circulating HMGB-1 with Induction-Treatment Response in Acute Myeloid Leukemia. In this cohort, lower baseline circulating HMGB-1 levels were associated with a reduced probability of successful induction response. The proposed model provides a biologically plausible framework for interpreting this association, integrating evidence from previous experimental studies on extracellular HMGB-1, TLR4/RAGE signaling, dendritic-cell activation, and immunogenic cell death. As HMGB-1 was measured at baseline, these pathways are presented as a conceptual interpretation of the observed association and warrant further mechanistic validation.

During immunogenic cell death, extracellular HMGB-1 released from damaged or dying malignant cells may activate TLR4- and RAGE-related signaling in antigen-presenting cells. ^13^ ^–^ ^15^ This process may promote dendritic-cell activation, antigen processing, and adaptive antitumor immune responses. ^21^ ^,^ ^29^ ^,^ ^32^ ^–^ ^34^ Therefore, higher circulating HMGB-1 could hypothetically reflect spontaneous leukemic-cell turnover or subclinical cell death and an immune microenvironment that is more responsive to subsequent cytotoxic treatment. ^13^ ^–^ ^15^ The proposed model presented in Figure 3 illustrates this possible link between leukemic-cell stress, extracellular HMGB-1 signaling, immune activation, and induction-treatment response.

However, HMGB-1 was measured before initiation of chemotherapy. Therefore, the measured concentration cannot be interpreted as HMGB-1 released directly in response to chemotherapy-induced leukemic-cell death. Baseline circulating HMGB-1 may reflect spontaneous leukemic-cell turnover, inflammatory activity, tissue injury, immune-cell activation, or other host- and disease-related processes. Consequently, the proposed relationship between baseline HMGB-1, immunogenic cell death, and subsequent chemotherapy response remains biologically plausible but was not directly demonstrated in this study.

The findings support further investigation of circulating HMGB-1 as an accessible biomolecular marker associated with early induction response in AML. Nevertheless, HMGB-1 should currently be considered an exploratory complementary biomarker rather than a substitute for established clinical assessment, cytogenetic and molecular risk classification, or measurable residual disease evaluation.

This study has several limitations. First, although patients were recruited at two national referral hospitals, the final biomarker-evaluable cohort was relatively small. The 71 included patients exceeded the minimum calculated sample size of 66. Second, excluding patients who died before post-induction response assessment may have introduced survivor-selection bias. Third, HMGB-1 was measured only once before induction chemotherapy; therefore, changes during and after treatment could not be evaluated.

Fourth, the assay measured total circulating HMGB-1 without distinguishing its cellular source or redox isoforms. Fifth, the HMGB-1 cut-off was derived and evaluated in the same cohort and therefore requires external validation. Sixth, comprehensive cytogenetic and molecular risk profiling was not systematically available. Finally, OS and EFS could not be estimated because complete dates of death, relapse, and last follow-up were unavailable. The findings should therefore be interpreted as associations with early induction response rather than evidence of the long-term prognostic significance of HMGB-1.

Despite these limitations, this study provides prospective biomarker data from an Indonesian adult AML population. Larger multicenter prospective studies incorporating serial HMGB-1 measurements, HMGB-1 redox-state characterization, comprehensive molecular profiling, measurable residual disease assessment, OS, EFS, and mechanistic validation are required to confirm these findings and define their clinical relevance.

## Conclusion

Low baseline circulating HMGB-1 was associated with a lower probability of successful induction response in the biomarker-evaluable adult AML cohort. This was consistent with the final clinical-biomolecular model, where high baseline HMGB-1 remained associated with successful response after adjustment for platelet count and ATR. Baseline HMGB-1 showed no significant correlation with bone marrow or peripheral blood blast percentages, miR-181a-3p, miR-181b-5p, ATM, or ATR.

As HMGB-1 was measured before chemotherapy, these findings reflect an association between baseline levels and early induction response rather than evidence of chemotherapy-induced HMGB-1 release or immunogenic cell death. Circulating HMGB-1 may serve as a complementary exploratory biomarker of early treatment response in AML, but its clinical utility requires external validation, serial measurements, comprehensive molecular risk assessment, and long-term outcome evaluation.

## Ethical considerations

This study was conducted in accordance with the principles of the Declaration of Helsinki. The parent prospective study from which this manuscript was derived received ethical approval from the Medical Research Ethics Committee of the Faculty of Medicine, Universitas Indonesia – Dr. Cipto Mangunkusumo National General Hospital (Approval No. KET-104/UN2.F1/ETIK/PPM.00.02/2025; approved on 7 February 2025).

As the study was conducted collaboratively across multiple study sites, including Dharmais National Cancer Center Hospital and Dr. Cipto Mangunkusumo National General Hospital, the research was also registered and acknowledged by the Health Research Ethics Committee of Dharmais Cancer Hospital (Notification No. DP.04.03/11.7/076/2025; dated 4 March 2025). The present manuscript represents a secondary biomarker analysis derived from the approved parent study. Written informed consent was obtained from all participants prior to enrolment.

## Data Availability

The datasets generated and analysed during the current study contain individual-level clinical and laboratory information from patients with acute myeloid leukemia. Public deposition of these data is restricted due to ethical and privacy considerations and in accordance with the conditions approved by the Health Research Ethics Committee of Dr. Cipto Mangunkusumo National General Hospital, Faculty of Medicine Universitas Indonesia. The Ethics Committee approved the study on the condition that participant confidentiality and privacy are protected. Therefore, raw patient-level data cannot be made publicly available. Researchers who wish to access de-identified data for legitimate academic purposes may submit a written request to the corresponding author ( restimulyasari@gmail.com). Requests will be reviewed by the corresponding author and the relevant institutional authorities. Access may be granted following ethical review, execution of a data-sharing agreement, and confirmation that the proposed use is consistent with the original informed consent and applicable regulations.
