# Supplementary material for: Baseline Extracellular HMGB-1 Levels Associated with Induction Chemotherapy Response in Adult Acute Myeloid Leukemia
Source: F1000Res. 2026 Aug 18;15:1143. Originally published 2026 Jul 11. [Version 2] doi: 10.12688/f1000research.183970.2 (PMC13562947; doi:10.12688/f1000research.183970.2)
Supplement: Peer review history

# Reviewer report: Reviewer response for version 2

**DOI:** 10.5256/f1000research.207777.r512299

**Published:** 2026-09-02

**Authors:** Harsh Goel

**Affiliations:** 1 All India Institute of Medical Sciences, New Delhi, India

*I have reviewed the revised manuscript and the authors’ responses to my previous comments. In my opinion, the comments and concerns raised during the peer review process have been appropriately addressed and incorporated into the revised version. The authors have made substantial improvements to the clarity and reporting of the study, including the methodology, results, interpretation of the findings, and discussion of the study’s limitations.*

*Overall, I am satisfied with the revisions, and I believe the revised manuscript is suitable for indexing. Therefore, I would be happy to amend my previous status from 'Approved with Reservations' to 'Approved'.*

Is the work clearly and accurately presented and does it cite the current literature?

Partly

If applicable, is the statistical analysis and its interpretation appropriate?

Partly

Are all the source data underlying the results available to ensure full reproducibility?

Partly

Is the study design appropriate and is the work technically sound?

Partly

Are the conclusions drawn adequately supported by the results?

Partly

Are sufficient details of methods and analysis provided to allow replication by others?

Partly

Reviewer Expertise:

NA

I confirm that I have read this submission and believe that I have an appropriate level of expertise to confirm that it is of an acceptable scientific standard.

# Reviewer report: Reviewer response for version 2

**DOI:** 10.5256/f1000research.207777.r512300

**Published:** 2026-08-28

**Authors:** Deniz Ozmen

**Affiliations:** 1 Istanbul University Cerrahpaşa, Istanbul, Turkey

All the issues that have been pointed to by the reviewers have been addressed by the authors.

Is the work clearly and accurately presented and does it cite the current literature?

Yes

If applicable, is the statistical analysis and its interpretation appropriate?

Partly

Are all the source data underlying the results available to ensure full reproducibility?

Partly

Is the study design appropriate and is the work technically sound?

Partly

Are the conclusions drawn adequately supported by the results?

Yes

Are sufficient details of methods and analysis provided to allow replication by others?

Partly

Reviewer Expertise:

Hematology, leukemias, myeloid diseases

I confirm that I have read this submission and believe that I have an appropriate level of expertise to confirm that it is of an acceptable scientific standard.

# Reviewer report: Reviewer response for version 1

**DOI:** 10.5256/f1000research.203070.r502400

**Published:** 2026-07-28

**Authors:** Deniz Ozmen

**Affiliations:** 1 Istanbul University Cerrahpaşa, Istanbul, Turkey

**1. Serial Measurements and Baseline Limitations:**

 Measuring biomarkers solely at baseline simplifies the study design and limits its scope. It would have been methodologically stronger to perform serial post-treatment measurements to assess dynamic changes in danger signaling molecules. Although the title correctly explicitly states that baseline levels were measured, the lack of serial measurements should be explicitly acknowledged as a study limitation in the Discussion section.

**2. Dual Role of HMGB-1:**

 It is positive that the authors addressed the dual biological role of HMGB-1, acknowledging that elevated HMGB-1 levels do not necessarily exert an immune-stimulating or anti-tumor effect in all contexts.

**3. Attrition and Survivor Bias (Excluded Deceased Patients):**

 Deceased patients were excluded from the final analysis, which creates a significant selection/survivor bias and limits the study's generalizability. The baseline HMGB-1 measurements for these patients should be reported (if available), and this high attrition rate must be listed as a major limitation in the Discussion.

**4. Reporting of Cut-off Values:**

 The authors used ROC analysis to categorize HMGB-1 levels, but the exact numerical cut-off value (e.g., in ng/mL) used to define "high" vs. "low" groups is missing. The specific ROC cut-off value, along with the Area Under the Curve (AUC), sensitivity, and specificity, must be clearly reported in the results.

**5. Nomenclature Consistency:**

 Please standardize the spelling/formatting of HMGB-1 throughout the manuscript (e.g., choose consistently between "HMGB-1" and "HMGB1").

Is the work clearly and accurately presented and does it cite the current literature?

Yes

If applicable, is the statistical analysis and its interpretation appropriate?

Partly

Are all the source data underlying the results available to ensure full reproducibility?

Partly

Is the study design appropriate and is the work technically sound?

Partly

Are the conclusions drawn adequately supported by the results?

Yes

Are sufficient details of methods and analysis provided to allow replication by others?

Partly

Reviewer Expertise:

Hematology, leukemias, myeloid diseases

I confirm that I have read this submission and believe that I have an appropriate level of expertise to confirm that it is of an acceptable scientific standard, however I have significant reservations, as outlined above.

# Response

**Published:** 2026-07-31

**Authors:** Resti Mulya Sari

**Affiliations:** Hematology & Medical Oncology, Dharmais Cancer Hospital, West Jakarta, Jakarta, Indonesia

1. **Response to Reviewer 2 (Deniz Ozmen, Istanbul University Cerrahpaşa, Istanbul, Turkey)**

We sincerely thank the Reviewer for the careful assessment of our manuscript and for the constructive recommendations. We are grateful that the Reviewer recognized our discussion of the dual biological role of HMGB-1. We have addressed each additional comment as follows.

**Comment 1: Serial Measurements and Baseline Limitations**

*“Measuring biomarkers solely at baseline simplifies the study design and limits its scope. It would have been methodologically stronger to perform serial post-treatment measurements to assess dynamic changes in danger signaling molecules. Although the title correctly explicitly states that baseline levels were measured, the lack of serial measurements should be explicitly acknowledged as a study limitation in the Discussion section.”*

**Response:**

 We fully agree. HMGB-1 and the other biomarkers were measured only once before induction chemotherapy. Therefore, the present study could not evaluate dynamic changes during or after treatment or determine whether chemotherapy resulted in additional HMGB-1 release.

 We have explicitly added this issue to the limitations in the Discussion. We also clarified throughout the manuscript that the results concern baseline circulating HMGB-1 and should not be interpreted as direct evidence of chemotherapy-induced HMGB-1 release or immunogenic cell death.

 The revised Discussion further recommends serial measurements before, during, and after induction chemotherapy in future prospective studies. We agree that such a design would be necessary to assess temporal biomarker changes and their relationship with leukemic-cell death, inflammatory signaling, treatment response, relapse, and survival.

**Comment 2: Dual Role of HMGB-1**

*“It is positive that the authors addressed the dual biological role of HMGB-1, acknowledging that elevated HMGB-1 levels do not necessarily exert an immune-stimulating or anti-tumor effect in all contexts.”*

**Response:**

 We thank the Reviewer for this positive assessment. We retained and further strengthened this discussion.

 The revised manuscript explains that HMGB-1 activity depends on its cellular localization, mode of release, post-translational modification, redox state, receptor engagement, and biological context. Intracellular HMGB-1 may support autophagy, cell survival, and chemotherapy resistance, whereas extracellular HMGB-1 may act as a damage-associated molecular pattern. Nevertheless, extracellular HMGB-1 can also promote inflammation, survival signaling, migration, and tumor progression.

 We therefore emphasize that an elevated total circulating HMGB-1 concentration cannot automatically be interpreted as evidence of an immune-stimulatory or antitumor effect. The mechanistic interpretation remains exploratory.

**Comment 3: Attrition and Survivor Bias**

*“Deceased patients were excluded from the final analysis, which creates a significant selection/survivor bias and limits the study's generalizability. The baseline HMGB-1 measurements for these patients should be reported (if available), and this high attrition rate must be listed as a major limitation in the Discussion.”*

**Response:**

 We agree that the exclusion of patients who died before standardized post-induction response assessment represents an important source of potential survivor-selection bias.

 Among 102 patients who initiated induction chemotherapy, 25 died during induction or early post-induction care. Because these patients did not undergo the standardized post-induction bone marrow assessment required to classify the study outcome, they could not be included in the response analysis. Complete evaluable baseline HMGB-1 measurements linked to standardized post-induction outcomes were not available for this deceased group; therefore, a reliable comparison of baseline HMGB-1 according to induction mortality could not be performed.

 We have now:

- reported the 25 deaths among the 102 treated patients;
- described the complete patient-selection and attrition process in the Methods and Results;
- clarified that the final 71 patients constitute a biomarker-evaluable cohort;
- added a patient-flow diagram;
- identified survivor-selection bias as a major study limitation; and
- cautioned that the observed response proportion should not be interpreted as the overall effectiveness of induction chemotherapy among all treated patients.

We agree that this attrition limits the generalizability of the findings, particularly to patients who die before completing induction-response assessment.

**Comment 4: Reporting of Cut-off Values**

*“The authors used ROC analysis to categorize HMGB-1 levels, but the exact numerical cut-off value (e.g., in ng/mL) used to define ‘high’ vs. ‘low’ groups is missing. The specific ROC cut-off value, along with the Area Under the Curve (AUC), sensitivity, and specificity, must be clearly reported in the results.”*

**Response:**

 Thank you for identifying this reporting issue. We have expanded the Methods and Results to provide the complete ROC information.

 When successful induction response was coded as the positive outcome, baseline circulating HMGB-1 yielded an AUC of 0.615 (95% CI, 0.480–0.749). The Youden index identified an exploratory optimal cut-off of 28.59 pg/mL, with a sensitivity of 65.0% and a specificity of 61.3%.

 HMGB-1 concentrations ≤28.59 pg/mL were categorized as low, whereas concentrations >28.59 pg/mL were categorized as high. These values are now reported consistently in the Abstract, Methods, Results, and relevant tables.

 We also added the ROC curve as Figure 2. Because the cut-off was derived and evaluated in the same cohort and the discriminatory performance was modest, we now explicitly describe the threshold as exploratory and emphasize that it requires external validatio

# Reviewer report: Reviewer response for version 1

**DOI:** 10.5256/f1000research.203070.r502396

**Published:** 2026-07-22

**Authors:** Harsh Goel

**Affiliations:** 1 All India Institute of Medical Sciences, New Delhi, India

1. Prevalence data of AML among Indonesian population in introduction section is too old. Kindly revise the latest data.

 2. Please revise the introduction include the OS, EFS of AML, factors responsible for the disease as well.

 3. Describe some ELN classification of AML in the introduction section.

 4. Explain the molecular mechanism of HMGB1 and its role in AML.

 5. How you have calculated the sample size for this study.

 6. Why pediatric patients were excluded from this study. AML is also observed among children.

 7. Correlate the clinicopathological characteristics of AML with the expression of HMGB1.

 8. Kindly perform univariate and multivariate analysis to find the prognostic significance of HMGB1.

 9. Make one table showing detailed clinical characteristics including blast count, molecular markers, immunophenotype of AML included in this study.

 10. You have not Estimated OS and EFS among your patients. Kindly correlate is the expression of HMGB1 is linked with survival.

 11. Kindly improve the discussion by comparing the outcome of your data with already published study in AML.

 12. This study seems to be reanalyzed as the results were not properly correlate with AML.

Is the work clearly and accurately presented and does it cite the current literature?

Partly

If applicable, is the statistical analysis and its interpretation appropriate?

Partly

Are all the source data underlying the results available to ensure full reproducibility?

Partly

Is the study design appropriate and is the work technically sound?

Partly

Are the conclusions drawn adequately supported by the results?

Partly

Are sufficient details of methods and analysis provided to allow replication by others?

Partly

Reviewer Expertise:

Hematological Malignancies

I confirm that I have read this submission and believe that I have an appropriate level of expertise to confirm that it is of an acceptable scientific standard, however I have significant reservations, as outlined above.

# Response

**Published:** 2026-07-31

**Authors:** Resti Mulya Sari

**Affiliations:** Hematology & Medical Oncology, Dharmais Cancer Hospital, West Jakarta, Jakarta, Indonesia

1. **Response to Reviewer 1 (Harsh Goel, All India Institute of Medical Sciences, New Delhi, India)**

We sincerely thank the Reviewer for the comprehensive and constructive assessment of our manuscript. We have carefully considered each comment and revised the manuscript accordingly.

**Comment 1: Epidemiological information and AML prevalence in Indonesia**

**Response:**

 Thank you for highlighting the need for more appropriate epidemiological context. We revised the Introduction using updated Global Cancer Observatory data for Indonesia. Because the available national estimates combine all leukemia subtypes, we explicitly clarified that these figures should not be interpreted as AML-specific national incidence or prevalence data.

 We also added a contemporary Indonesian hospital-based AML cohort reported by Hadisantoso et al. This study provides relevant information regarding adult patients with AML treated with standard 7+3 induction chemotherapy at two Indonesian national referral hospitals. The distinction between national leukemia estimates and hospital-based AML data is now clearly stated.

**Comment 2: Overall survival and event-free survival**

**Response:**

 We agree that overall survival (OS) and event-free survival (EFS) are important clinical outcomes in AML. However, OS and EFS were not prespecified outcomes of this study, and the available biomarker dataset did not contain sufficiently complete dates of death, relapse, and last follow-up to support reliable time-to-event analyses.

 Consequently, Kaplan–Meier and Cox proportional-hazards analyses could not be performed without introducing potentially misleading estimates. We have explained this issue in the Methods, Results, and Discussion. We also clarified that the present study evaluates associations with early post-induction response and does not establish the long-term prognostic significance of HMGB-1.

**Comment 3: Clinical and disease-related factors influencing AML outcomes**

**Response:**

 Thank you for this recommendation. The Introduction was expanded to acknowledge that AML outcomes are influenced by age, performance status, comorbidities, disease ontogeny, leukocyte and blast counts, cytogenetic and molecular abnormalities, treatment intensity, induction response, and measurable residual disease.

 The manuscript now also discusses established factors associated with AML development, including increasing age, antecedent myelodysplastic or myeloproliferative neoplasms, inherited cancer-predisposition syndromes, previous cytotoxic chemotherapy or radiotherapy, previous cytotoxic chemotherapy or radiotherapy, benzene exposure, and cigarette smoking.

**Comment 4: ELN risk classification and molecular characterization**

**Response:**

 We agree that contemporary AML assessment should incorporate the European LeukemiaNet risk classification. We therefore added a description of the ELN 2022 risk categories in the Introduction and clarified the importance of cytogenetic and molecular abnormalities in AML prognosis.

 However, cytogenetic and comprehensive molecular data were not available in this study due to technical and resource limitations, and therefore could not be assessed. As a result, ELN risk stratification could not be performed in the study cohort.

 We have transparently reported this limitation in the Methods, Results, and Discussion and avoided interpreting untested molecular abnormalities as negative findings.

**Comment 5: Sample-size calculation**

**Response:**

 Thank you for requesting clarification of the sample-size calculation. We added the diagnostic-sensitivity formula to the Methods:

*Nsen = Zα² × Sen(1−Sen)/(d² × P)*

 The calculation assumed an expected biomarker sensitivity of 70%, an anticipated prevalence of successful induction response of 55%, a precision of 15%, and a two-sided alpha level of 5%, corresponding to a Zα value of 1.96. The minimum calculated sample size was 66 participants. Ultimately, 71 patients had complete induction-response and biomarker data and were included in the final analysis, exceeding the minimum required sample size.

**Comment 6: Exclusion of pediatric patients**

**Response:**

 We have clarified that this prospective study was specifically designed for adults treated within adult hematology-oncology services. Pediatric AML differs substantially from adult AML in its cytogenetic and molecular landscape, treatment protocols, chemotherapy dose intensity, supportive-care requirements, treatment-related toxicity, and long-term outcomes.

 Combining pediatric and adult populations could therefore introduce considerable biological and therapeutic heterogeneity. This justification has been added to the Study Population subsection.

**Comment 7: Patient selection, induction mortality, and evaluable cohort**

**Response:**

 We agree that the patient-selection process required more transparent reporting. We have revised the Methods and Results and added a patient-flow diagram.

 Initially, 109 adults with newly diagnosed AML were assessed for eligibility. Of the 102 patients who initiated induction chemotherapy, 25 died during induction or early post-induction care. Two additional patients were lost to follow-up, and four patients had non-evaluable biomarker results. The final biomarker-evaluable cohort therefore comprised 71 patients.

 We now explicitly state that the 71-patient cohort represents an evaluable-patient biomarker cohort rather than the entire population initiating induction chemotherapy. The induction mortality of 25 among 102 treated patients is reported separately. The potential survivor-selection bias resulting from exclusion of patients who died before standardized response assessment is acknowledged as a major limitation.

**Comment 8: Definition of treatment response**

**Response:**

 Thank you for identifying the need for greater precision in the outcome terminology. The favorable-response category in the parent study included morphological leukemia-free state, complete remission, complete remission with partial hematologic recovery, and complete remission with incomplete hematologic recovery. Partial remission and absence of response were categorized as unsuccessful responses.

 Because this composite endpoint extends beyond conventional complete remission, we standardized the terminology by using the term “successful induction response” when referring to the combined favorable-response group throughout the manuscript. However, the term “complete remission (CR)” was retained where it refers specifically to the established clinical definition in accordance with standard AML response criteria. The response definitions and post-induction assessment procedures are now clearly described in the Methods.

**Comment 9: Baseline characteristics and biomolecular data**

**Response:**

 We revised the Results tables to provide a clearer presentation of the available clinical, hematologic, morphologic, and biomolecular data. Table 1 summarizes age, sex, FAB subtype, and induction-response status. Table 2 presents clinical and laboratory characteristics according to successful induction-response status, whereas Table 3 presents the bivariable associations between the evaluated baseline biomarkers and successful induction response. Table 4 reports the correlations between baseline HMGB-1 and the evaluated morphologic and biomolecular characteristics, and Table 5 presents the initial and final clinical-biomolecular models.

 Complete patient-level immunophenotypic, cytogenetic, and molecular profiles were not systematically available and therefore could not be comprehensively analyzed.

**Comment 10: Association between HMGB-1 and clinicopathological characteristics**

**Response:**

 We added Spearman correlation analyses examining baseline circulating HMGB-1 in relation to bone marrow blast percentage, peripheral blood blast percentage, miR-181a-3p, miR-181b-5p, ATM, and ATR.

 No statistically significant correlations were identified. The correlations were weak, indicating that baseline circulating HMGB-1 was not detectably associated with morphologic blast burden or the other evaluated baseline biomarkers in this cohort. Specifically, the correlation between baseline HMGB-1 and ATR was weak and nonsignificant (Spearman’s r = 0.148; p = 0.217; n = 71). These findings are now presented in a dedicated Results table and interpreted cautiously in the Discussion.

**Comment 11: Bivariable and multivariable analyses**

**Response:**

 We revised the statistical reporting to distinguish the bivariable and multivariable analyses clearly. Variables meeting the predefined criterion of p<0.25 in the bivariable analysis were considered for inclusion in the multivariable clinical-biomolecular model.

 In the bivariable analysis, low baseline HMGB-1 (≤28.59 pg/mL) was associated with a lower probability of successful induction response than high baseline HMGB-1 (RR 0.620; 95% CI 0.394–0.975; p=0.038). The initial multivariable model included platelet count >22,500/µL, serum creatinine >0.74 mg/dL, high HMGB-1 >28.59 pg/mL, and high ATR >29.16 pg/mL. After backward elimination, platelet count, HMGB-1, and ATR were retained in the final model. In the complementary high-versus-low coding used in this model, high baseline HMGB-1 remained associated with a higher probability of successful induction response after adjustment for platelet count and ATR.

 Thus, the bivariable and multivariable findings were directionally consistent: low baseline HMGB-1 was associated with a lower probability of successful response, whereas high baseline HMGB-1 was associated with a higher probability of successful response. The initial and final models are now presented separately with their effect estimates, 95% confidence intervals, and p-values.

**Comment 12: Interpretation of the dual biological role of HMGB-1**

**Response:**

 We appreciate the Reviewer’s concern regarding the apparently contradictory biological roles of HMGB-1. We substantially revised the Discussion to explain that HMGB-1 functions as a context-dependent or “double-edged” molecule.

 Intracellular and cytoplasmic HMGB-1 may promote leukemic-cell survival, autophagy, inhibition of apoptosis, and chemotherapy resistance. In contrast, extracellular HMGB-1 released from damaged or dying malignant cells may function as a damage-associated molecular pattern. Its biological activity also depends on post-translational modification and redox state. Fully reduced HMGB-1 primarily exhibits chemotactic activity, disulfide HMGB-1 can induce cytokine signaling through the TLR4/MD-2 complex, and terminal oxidation may abolish these activities.

 Accordingly, we now explicitly state that a higher total circulating HMGB-1 concentration cannot automatically be interpreted as evidence of an immune-stimulatory or anti tumor effect.

**Comment 13: Biological plausibility of the TLR4- and RAGE-related mechanism**

**Response:**

 Thank you for encouraging us to provide stronger evidence and a more balanced interpretation of the proposed mechanism. We revised the Introduction, Discussion, Figure 3, and its legend and added supporting references concerning the context-dependent biological activities of extracellular HMGB-1.

 Experimental evidence indicates that extracellular HMGB-1 released during immunogenic tumor-cell death may interact with TLR4 on dendritic cells and support tumor-antigen processing and subsequent T-cell priming. We also clarified that RAGE represents an additional context-dependent HMGB-1 signaling pathway rather than an exclusively antitumor pathway, because RAGE signaling may contribute to inflammation, cell survival, migration, and tumor progression.

 Importantly, Figure 3 is now explicitly described as a literature-based, hypothesis-generating conceptual model. The figure distinguishes the observed clinical finding—low baseline circulating HMGB-1 was associated with a lower probability of successful induction response—from the proposed post-treatment pathway, which was not directly examined in the present study. We also state that HMGB-1 redox isoforms, chemotherapy-induced HMGB-1 release, TLR4 and RAGE activation, dendritic-cell function, and immunogenic cell death were not measured.

**Comment 14: Baseline HMGB-1 versus chemotherapy-induced HMGB-1 release**

**Response:**

 We fully agree that this distinction is essential. HMGB-1 was measured before initiation of induction chemotherapy. Therefore, the measured concentration cannot be interpreted as HMGB-1 released directly in response to chemotherapy-induced leukemic-cell death.

 We revised the Abstract, Introduction, Discussion, and Conclusion to clarify that baseline circulating HMGB-1 may reflect spontaneous leukemic-cell turnover, inflammatory activity, tissue injury, immune-cell activation, or other host- and disease-related processes.

 The proposed relationship between HMGB-1, immunogenic cell death, TLR4/RAGE signaling, and chemotherapy response is now presented only as a biologically plausible mechanism supported by previous experimental studies. We explicitly state that post-treatment HMGB-1 release, receptor activation, dendritic-cell function, and immunogenic cell death were not directly measured in this study.

**Comment 15: Conclusions and clinical implications**

**Response:**

 We revised the Conclusion to maintain the same exposure orientation as the primary categorical analysis and Figure 3. The manuscript now concludes that low baseline circulating HMGB-1 was associated with a lower probability of successful induction response in the biomarker-evaluable cohort.

 We intentionally used the term “associated with” rather than causal language such as “caused” or “reduced,” because the observational design does not establish causality. We also clarified that the Spearman analyses addressed correlations between HMGB-1 and morphologic or biomolecular characteristics and were not the basis of the primary treatment-response conclusion.

 HMGB-1 is described as a complementary exploratory biomarker rather than a replacement for established clinical assessment, cytogenetic and molecular risk classification, or measurable residual disease evaluation. We also emphasize the need for external validation, serial biomarker measurements, comprehensive molecular profiling, and long-term outcome assessment.
